# Marketplace Analysis Demonstrates Quality Control Standards Needed for Black Raspberry Dietary Supplements

**DOI:** 10.1007/s11130-014-0416-y

**Published:** 2014-04-25

**Authors:** Jungmin Lee

**Affiliations:** United States Department of Agriculture, Agricultural Research Service, Horticultural Crops Research Unit Worksite, 29603 U of I Ln., Parma, ID 83660 USA

**Keywords:** Authenticity, Adulteration, Pigment, *Rubus*, *Rubus occidentalis* L, Black cap

## Abstract

There is currently no standard for the minimum anthocyanin concentration a black raspberry dietary supplement must contain for legal sale in the US. All consumer available black raspberry products (*n* = 19), packaged as dietary supplements or otherwise prepared (freeze-dried whole and pre-ground powders), were purchased and analyzed for their anthocyanin composition and concentration. Seven of the 19 samples contained no anthocyanins from black raspberry fruit, while three of those seven (without black raspberry fruit) had no anthocyanins of any kind. There was a wide range of anthocyanin concentration within the remaining products (18.1–2,904.8 mg/100 g; *n* = 12). When expressed as per capsule or per ∼1 teaspoon, concentration ranged from 0.1 to 145.2 mg (average 28 mg; *n* = 12). Until US dietary supplement labeling comes under regulatory oversight similar to food guidelines, foods are a more dependable source for dietary phenolics than supplements.

## Introduction

The USDA (United States Department of Agriculture) has put together several campaigns advocating a healthy eating diet by having one that is color diverse (USDA 5 A Day campaign, USDA ChooseMyPlate.gov, HealthierUS School Challenge-HUSSC, etc.). As consumers have become increasingly aware of the benefits from eating healthier, sales of black raspberry supplements have also increased, and were further raised after a popular US media program promoted black raspberry consumption for its cancer fighting potential. This popular television show made the assertion that an adult should consume 600 mg of anthocyanins from black raspberry dietary supplements daily (300 mg twice a day), followed by the unreasonable claim that each 300 mg capsule (featured product) contained the equivalent content of four cups of fresh black raspberries. Two samples linked with the group making these statements were examined in this study, and are discussed later. Although the potential health benefits of black raspberry fruit and its specific mechanisms are still under investigation [1–3], unwitting consumer demand has increased the availability of products described as black raspberry supplements in the dietary supplement marketplace.

Black raspberry (*Rubus occidentalis* L.; native to Eastern North America) fruit has been traditionally used as a food and a natural colorant, but renewed US consumer interest has brought an upsurge in the number of commercial black raspberry products available (from desserts to dietary supplements; [4]). This can partially be explained by increased awareness of the potential health benefits high-pigmented fruit might provide [1–3, 5, 6], but their distinct flavor, unlike blackberries or red raspberries, may also help. Unfortunate side effects of intensified consumer demand have been occurrences of product adulteration, though some likely arose from the limited production of black raspberries, which due to their unique growing requirements make Oregon the only US state with notable acreage [4].

Black raspberry (*Rubus occidentalis* L.) has fruit, plant phenology, plant morphology, and anthocyanin profile distinctly different from red raspberry, blackberry, or any other genus *Rubus* berries [5, 7–9]. Since most people are unable to distinguish black raspberries, a simple fact sheet has been generated (www.black-raspberry.com) to help educate researchers, health professionals, industry, and consumers the differences among black raspberry, red raspberry, and blackberry. It is apparent that black raspberry dietary supplement producers and sellers might not be able to differentiate between black raspberry and blackberry, as the images on the supplements’ packaging were incorrect in four of the products examined in this study (summarized in Table 1). The inaccurate images used were that of either blackberries or an altered image of blackberries (white core was colored in black). The confusion about black raspberry fruit’s correct morphology [8, 9], and the sudden boost in available black raspberry products (although the fruit production is limited; [10]), caused us to examine the anthocyanin concentration of marketplace black raspberry dietary supplements, and other forms that can be used as supplements (*i.e.*, freeze-dried black raspberries, extracts).

**Table 1:** Sample codes, brief summary of label information (codes used in Table 2), and observations. All samples were labeled on the bottle or package that contained black raspberry

Sample code	Information provided on product packaging. Cost per capsule or teaspoon. Entire package price.	Observations and comments.
A01	500 mg of black raspberry fruit extract. $0.11/capsule. Total cost $6.30.	Misspelled *Rubus leucodermis* on bottle. Latin name used on label may be incorrect, as the majority of commercially grown black raspberries are *R. occidentalis* not *R. leucodermis* [10]. Contained unlisted non-fruit ingredients. Capsule content weight was 0.49 g.
A02	One capsule contains 425 mg of *R. occidentalis* berry. $0.17/capsule. Total cost $19.95.	Contained unlisted ingredients. Capsule content weight was 0.81 g.
A03	550 mg *Rubus occidentalis* berry. $0.13/capsule. Total cost $11.99.	Label misspelled ‘berry’. Contained unlisted ingredients besides dried fruit powder. Label had correct black raspberry image. Capsule content weight was 0.48 g.
A04	100 % pure black raspberry dietary supplement. 400 mg of black raspberry powder per capsule. Vegetable capsule (plant derived cellulose). $0.31/capsule. Total cost $18.39.	Capsule content did not appear to be pure black raspberry fruit powder. Capsule content weight was 0.46 g. Contained unlisted non-fruit ingredients.
A05	One capsule contains 250 mg of black raspberry. $0.15/capsule. Total cost $18.34.	Blackberry image on label. Contained unlisted non-fruit ingredients besides dried fruit powder. Capsule content weight was 0.61 g.
A06	300 mg of black raspberry (as *Rubus occidentalis* berry). $0.18/capsule. Total cost $16.05.	Contained unlisted non-fruit ingredients besides dried fruit powder. Capsule content weight was 0.30 g.
A07	300 mg of *Rubus occidentalis* fruit. Freeze-dried. $0.15/capsule. Total cost $8.99.	Blackberry image on label. Capsule content weight was 0.32 g. Capsule contents appeared to be pure black raspberry fruit powder.
A08	Each capsule contains 300 mg of black raspberry (*Rubus occidentalis*). Freeze-dried fruit. $0.14/capsule. Total cost $12.95.	Capsule content weight was 0.32 g. Capsule contents appeared to be pure black raspberry fruit powder.
A09	Clinical strength black raspberry extract. Contains 6 % alcohol. Anthocyanin equivalent to ∼500 fresh black raspberries or 100 g of freeze-dried powder. Fruit grown in Oregon. $2.50/teaspoon. Total cost $29.99.	Only liquid (extract) form available. Company claims clinical strength based on Stoner et al. [1] work. Description indicated a juice concentrate with small amount of alcohol. Appeared as a highly viscous liquid, like a juice concentrate. Label had correct black raspberry image.
A10	One 425 mg capsule has the micronutrient equivalent of over 4 cups of fresh berries. $0.11/capsule. Total cost $6.50.	Blackberry image on label. No 520 nm absorbing compounds. Contained unlisted non-fruit ingredients besides dried fruit powder. May have included colored filler. Capsule content weight was 0.54 g.
A11	Fresh, raw, pure. 400 mg of seedless black raspberry powder. Vegetarian capsules and absolutely nothing else. $0.79/capsule. Total cost $23.77.	Modified blackberry image on label with the white core was blacked out. Dark olive-brown-black powder in capsule did not look like berry powder and had a medicinal odor. No 520 nm absorbing compounds. This sample contained no black raspberry fruit. Capsule content weight was 0.41 g. See photo image in Fig. 1.e.
A12	425 mg of black raspberry fruit. $0.18/capsule. Total cost $10.95.	Contained 520 nm absorbing compounds, but not black raspberry anthocyanins. Contained unlisted non-fruit ingredients. May have included colored filler. Capsule content weight was 0.51 g.
A13	425 mg *Rubus occidentalis* fruit. $0.06/capsule. Total cost $3.13.	No 520 nm absorbing compounds. Contained unlisted non-fruit ingredients besides. Capsule content weight was 0.47 g.
A14	300 mg black raspberry. Made in USA. $0.18/capsule. Total cost $15.95.	Contained 520 nm absorbing compounds, but not black raspberry anthocyanins. Only sample in opaque capsules. Contained unlisted non-fruit ingredients. Capsule content weight was 0.57 g.
A15	100 % vegetarian, black raspberry, pure *Rubus occidentalis* extract. 500 mg of extract per capsule. $0.23/capsule. Total cost $23.14.	Contained 520 nm absorbing compound (very early eluting compound), but not black raspberry anthocyanins. Contained unlisted non-fruit ingredients. Capsule content weight was 0.65 g.
B01	Freeze-dried whole fruit. Vacuumed packed. USDA grade A. No preservatives. Grown in Oregon. 100 % pure Oregon grown black raspberry. $1.20/teaspoon. Total cost $23.99.	Freeze-dried whole fruit was in a Millard food bag and vacuumed packaged. Label had correct black raspberry image.
B02	Freeze-dried powder. USDA grade A. No preservatives. Grown in Oregon. 100 % pure Oregon grown black raspberry. $1.43/ teaspoon. Total cost $28.63.	Powder was in a Millard food bag and vacuumed packaged. Label had correct black raspberry image.
B03	Black raspberry powder dietary supplement. Instructions for supplementing fruit in meals. $1.02/teaspoon. Total cost $29.20.	Powder was in a packaged in a can. Label had correct black raspberry image.
B04	Live organic black raspberry. Instructions on how to consume as a dietary supplement. $2.38/ teaspoon. Total cost $49.99.	Contained compounds that absorb at 520 nm, but not black raspberry anthocyanins (see Fig. 2.). One side of the bag was transparent. This powder had a different hue than samples B02 and B03 (appeared to be not as intense red-purple).

## Materials and Methods

### Samples, Reagents, Chemical, and Standards

An effort to purchase all commercially available black raspberry supplements and dried fruit (powder and whole fruit forms) were made (*n* = 19) from May to July of 2013 (Amazon.com, Inc., Seattle, WA, USA). No purchased products were past their expiration or best use by date. These samples represented products from 17 companies. Products A09, B01, and B02 were from one company. The rest of the samples were from different companies. Sample information is summarized in Table 1. Dietary supplements in capsules or extract were coded A01 to A15, with one sample (A09) in liquid form. The four available dried fruit products were purchased and coded B01 to B04. One sample was freeze-dried whole fruit (B01); while the remaining dried samples were in powder form. Five capsules and their contents were weighed in triplicate to determine the weight of the powder within the capsules, and to convert our findings into per capsule. Capsule contents of A11 were suspicious (see Table 1) and a second example was purchased to double check that the original product was not random error.

All chemicals, reagents, and standards used in this study were analytical or HPLC grade from Sigma-Aldrich Chemical Co. (St. Louis, MO, USA). Cyanidin-3-glucoside was purchased from Polyphenols Laboratories AS (Sandnes, Norway).

### Extraction and Sample Preparation

All powder contents of the capsules were removed and stored at −75 °C until extraction. The one example of freeze-dried whole fruit (B01) was ground (using a coffee bean grinder, model K2M2; Braun GmbH, Kronberg, Germany) prior to storage and subsequent extraction. Each sample group’s collected powders were pooled and kept frozen until the start of chemical extraction. Samples were extracted and expressed as-is, since that represented the form of intended consumption. Powders (initially 1.5 g) were extracted with high purity water (initially 15 mL; Millipore Simplicity UV, Millipore Corp., Billerica, MA, USA) by sonication for 15 min, centrifuged 10 min at 4,000 rpm, then filtered (Millipore 0.45 μm Millex-FH syringe filter, Bedford, MA, USA) prior to injection onto the HPLC system [12, 13]. Solid to liquid extraction ratio had to be altered for samples that indicated very low to zero levels of anthocyanins (3.0 g powder extracted in 10 mL of water). A09 was diluted (1.5 g:15 mL high purity water; by weight due to its high viscosity) and put through the same process as the other samples (sonication, centrifugation, and filtration) prior to HPLC analysis. ‘Munger’ fruit extract was obtained from previous study [10, 11, 14, 15].

### HPLC (High Performance Liquid Chromatography) Condition for Individual Anthocyanin Separation

HPLC/DAD (diode array detector)/MS (mass spectrometry) was used for anthocyanin elution as described in detail in our previous work [13], except for the use of a longer analytical column [8]. Briefly, an Agilent HPLC 1100 (Agilent Technologies Inc., Palo Alto, CA, USA) was used for this investigation. Individual peaks were monitored at 520, 280, and 255 nm. Anthocyanins were expressed as cyanidin-3-glucoside (Polyphenols Laboratories AS, Sandnes, Norway). Anthocyanin peaks were identified by retention time, UV–VIS spectra, external standards (when available), verified fruit with known anthocyanin profiles, and prior published research [8, 10, 11, 14, 16–18]. Analyses were conducted in duplicate. Results were expressed as mg of cyanidin-3-glucoside/100 g of powder, mg of cyanidin-3-glucoside/capsule for samples coded A01–A08, or 5 g (∼1 teaspoon) for samples coded A09 and B01–B03. Peaks 2 and 3 peak areas were split at each apex, where it was not co-eluting, and multiplied by two to obtain total peak area prior to calculations.

## Results and Discussion

**Fig. 1:**
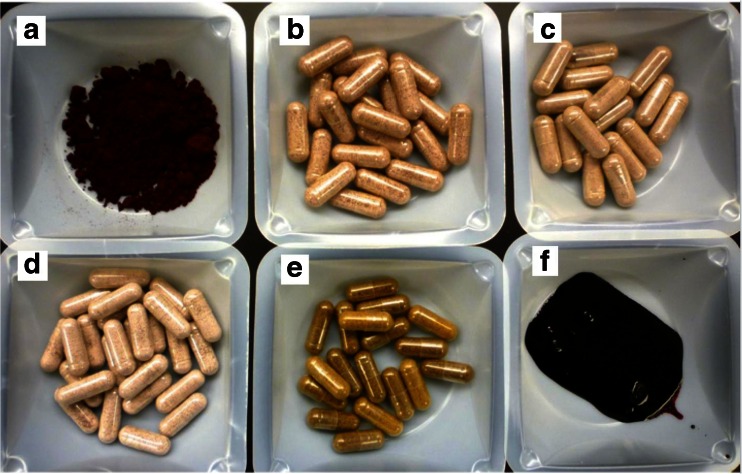
Photos of six black raspberry dietary supplements purchased. In photo example b through d, contained high amounts of filler. In photo example e (coded A11 in Table 1), contained no anthocyanin (no black raspberry fruit)

Relevant information from packaging, prices, and observations are summarized in Table 1; including the labeled contents from the products that were found to contain no black raspberry fruit. Photo examples of six dietary supplements can be found in Fig. 1. Supplements ranged in visual color from light pink to dark red, except for A11 capsules (see Fig. 1.e) that contained no red hue. An example of no filler added in the capsule contents (Fig. 1.a) compared to high amounts of filler used in Fig. 1.b through Fig. 1.d. Red appearance cannot be used to indicate the presence of black raspberry fruit since some of the manufacturers used pink colored fillers (A10 and A12) and other fruit powder (A12, A14, A15, and B04).

**Fig. 2:**
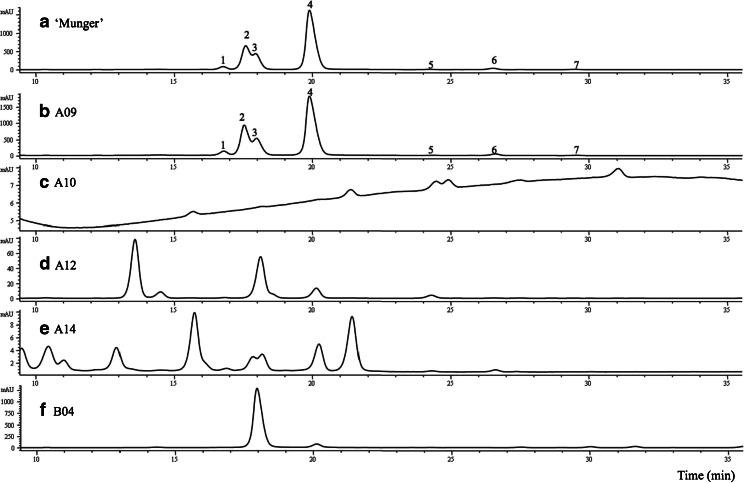
Anthocyanin profiles of black raspberries (a- ‘Munger’ and b- A09) and selected supplement samples (c- A10, d- A12, e- A14, and f- B04) that contained questionable materials other than black raspberry fruit. The chromatograms were monitored at 520 nm (280 and 255 nm traces not shown). Clearly, black raspberry anthocyanin profile is suitable for authenticity work (comparing ‘Munger’ to sample A09, leaves no doubt that A09 contains black raspberry). Corresponding peak identification for ‘Munger’ and A09 listed in Table 2. Additional black raspberry anthocyanin profiles can be found in our past work [7, 8, 10, 11]

Seven (A10–A15 and B04) of the 19 samples had no black raspberry anthocyanins. Out of those seven, A10, A11, and A13 contained no 520 nm absorbing compounds (chromatogram of A10 as an example in Fig. 2). Samples A12, A14, A15, and B04 contained 520 nm absorbing peaks, but their profiles did not match that of black raspberry (examples in Fig. 2.a and b). One anthocyanin profile, B04 from four freeze-dried samples (which were the B coded samples), did not match black raspberry either (see Fig. 2). We suspect B04 was blackberry (*Rubus* spp.; [5, 19]) freeze-dried powder and sold as black raspberry. Hydrochloric acid was used to confirm that A10, A11, and A13 contained no anthocyanins, as a color shift (redness due to oxonium formation) after acidification would also indicate the presence of anthocyanin [20–22], but this visual change did not occur in these three extracts. Some example chromatograms of these questionable materials are shown in Fig. 2 (c through f). Our second purchased A11 sample had an identical appearance (olive-brown-black powder; see Fig. 1.e) and the same HPLC profile (data not shown) as the first A11 sample, again with no detectable 520 nm absorbing peaks. Sample A03’s label claimed 550 mg of black raspberry in each capsule, though capsule entire content’s weight was measured at 480 mg (Table 1).

Cyanidin-3-rutinoside was the main anthocyanin among the twelve samples that did contain black raspberry fruit (Table 2). Six samples (A07, A08, A09, B01, B02, and B03) contained all seven anthocyanins routinely found in black raspberry fruit [5, 11, 14, 15]. These seven black raspberry anthocyanins in the order of elution are cyanidin-3-sambubioside, cyanidin-3-xylosylrutinoside, cyanidin-3-glucoside, cyanidin-3-rutinoside, pelargonidin-3-glucoside, pelargonidin-3-rutinoside, and peonidin-3-rutinoside. Six samples (A01 through A06) contained five of the seven anthocyanins, with only pelargonidin-3-glucoside and peonidin-3-rutinoside not being detected. Representative black raspberry fruit anthocyanin chromatograms can be found in Fig. 2 (a and b).

**Table 2:** Individual anthocyanin concentrations are listed in the order of HPLC elution (peak 1: cyanidin-3-sambubioside, peak 2: cyanidin-3-xylosylrutinoside, peak 3: cyanidin-3-glucoside, peak 4: cyanidin-3-rutinoside, peak 5: pelargonidin-3-glucoside, peak 6: pelargonidin-3-rutinoside, and peak 7: peonidin-3-rutinoside)

Sample code	Peak 1	Peak 2	Peak 3	Peak 4	Peak 5	Peak 6	Peak 7	Total (mg/100 g)	Total^a^ (mg/capsule or 5 g)
A01	0.4	3.0	2.1	12.4	nd^b^	0.2	nd	18.1 (0.1)	0.1 (0)
A02	0.4	5.7	1.3	14.1	nd	0.3	nd	21.6 (0.2)	0.2 (0.1)
A03	0.6	4.6	2.7	17.8	nd	0.2	nd	25.9 (0.1)	0.1 (0)
A04	0.6	8.0	2.9	23.8	nd	0.4	nd	35.7 (0.2)	0.2 (0)
A05	0.7	7.8	3.0	24.9	nd	0.3	nd	36.8 (0.2)	0.2 (0)
A06	3.3	7.6	17.7	85.5	nd	0.8	nd	114.8 (0.3)	0.3 (0)
A07	27.2	266.9	145.6	594.9	1.2	13.5	3.1	1052.4 (3.4)	3.4 (0.1)
A08	29.5	269.4	131.9	687.2	1.4	16.0	3.6	1138.8 (3.6)	3.6 (0.1)
A09	95.9	661.8	491.1	1606.5	4.9	37.4	7.3	2904.8 (38.5)	145.2 (1.9)
B01	18.2	223.4	74.2	496.0	0.8	13.4	2.6	828.5 (37.0)	41.4 (1.8)
B02	24.6	272.2	120.8	648.4	1.0	13.2	2.7	1082.8 (23.3)	54.1 (1.2)
B03	34.7	328.6	234.3	1114.6	1.8	21.8	6.5	1742.2 (85.9)	87.1 (4.3)

Total expressed as mg of cyanidin-3-glucoside/100 g and mg/capsule or 5 g (∼1 teaspoon). Values within parenthesis indicate standard errors. Seven samples (A10, A11, A12, A13, A14, A15, and B04) either contain no detectable anthocyanins or no black raspberry anthocyanins (see Table 1)

^a^A01–A08 were expressed as per capsule, A09 and B01–B03 as per ∼1 teaspoon (5 g)

^b^nd, not detected

Black raspberry labeled products (*n* = 19; Tables 1 and 2) contained zero to 2,904.8 mg/100 g of anthocyanin. Products that did contain black raspberry fruit anthocyanins ranged in concentration from 18.1 to 2,904.8 mg/100 g (*n* = 12; >160 fold difference), and 0.1 to 145.2 mg/capsule or ∼1 teaspoon (*n* = 12; >1,400 fold difference). The wide range in anthocyanins found in the dietary supplements are not surprising since our past work observed the fruit itself to vary from 3 to 996 mg/100 mL of fresh fruit (*n* > 1,000; [4, 10, 11, 14, 15]). But, a partial explanation for the wide ranges observed here were due to non-fruit ingredients (fillers, binders, bulking agents, carriers, etc.) such as rice powder, silica, magnesium stearate, etc. The black raspberry fruit containing capsules (A01–A08) averaged 305.5 mg/100 g or 1.0 mg/capsule, while A09 (the only sample in extract form) contained 2,904.8 mg/100 g or 145.2 mg/5 g (∼1 teaspoon). The black raspberry fruit containing freeze-dried products (B01–B03) averaged 1,217.9 mg/100 g or 60.9 mg/5 g (∼1 teaspoon). All samples were lower in anthocyanin than what had been previously reported for freeze-dried black raspberry powder: 3,200 mg/100 g (cultivar Jewel; [1]) and 4,360 mg/100 g (unknown cultivar; [23]). Possible contributors to these discrepancies include differences in sample handling (freeze-drying preparation, extraction, storage, etc.) and analysis conditions [5, 7].

A07 and A08 appeared to be pure fruit powders as indicated by their uniform dark red-purple powders (whole black raspberry seeds present) and high anthocyanin levels (A07- 1052.4 and A08- 1,138.8 mg/100 g). Based on observing A07 and A08, it would be possible to fill capsules without bulking agents and fillers as seen in the lower quality capsule samples. The highest anthocyanin concentration was from A09 (the liquid example) at 2,904.8 mg/100 g. If one consumed two capsules from sample A08 or A09, they ingested only ∼7.0 mg of anthocyanins. Samples B01, B02, and B03 if packaged as capsules, would provide amounts similar to A07 and A08, since those two capsule products contained 100 % freeze-dried berries.

Samples A04 and A11 are linked to the unnamed group mentioned in the introduction. A04 contained 0.2 mg/capsule, while A11 contained the mysterious olive-brown-black powder with a strange medicinal odor. A04 contained ingredients other than fruit powder that were not listed on the label (Table 1). In fact, three samples (A04, A06, and A11) did not indicate additional ingredients in capsules, but based on the visual appearance and HPLC anthocyanin results it is clear they did contain substances other than black raspberry fruit powder.

Using anthocyanin profile for identifying food and dietary supplement adulteration is not a new concept and has been demonstrated before [7, 8, 24, 25]. Misidentification of plant source material is a known issue in the US dietary/herbal supplement industry [26–29] and it is an obvious problem that needs to be corrected. It is possible that due to supply demand that the dietary supplement companies were fraudulent, or made honest mistakes from not testing constituents prior to production. Some issues surrounding dietary supplements can be resolved by improved dietary supplement regulations, and by endorsing proposed rules [30–34] for the safety of US consumers before another “ephedrine” scale incident occur [29].

## Conclusion

While there are companies (four companies herein) that provide consumers with high black raspberry anthocyanin containing products (A07, A08, A09, B01, B02, and B03), the majority (>70 %) of companies are selling low quality products, some containing unknown/unreported ingredients and very little black raspberry fruit. Until US dietary supplement products are better regulated and quality control standards for safety, purity, and dosage are defined and endorsed, the safer source for dietary phenolics as a consumer is from food intake [6]. From past research [24, 35, 36] and findings herein, there is a real need to create standards for dietary supplements made from plant sources. At the moment, a consumer who assumes the US dietary supplement marketplace is free from risk is unfortunately naive. Forty percent (seven out of 19) of the black raspberry supplements and products purchased and evaluated here contained no black raspberry fruit anthocyanin.
